# Life's Essential 8 cardiovascular health status of 18–69-year-old individuals in France

**DOI:** 10.1016/j.ajpc.2025.100981

**Published:** 2025-03-30

**Authors:** Omar Deraz, Sofiane Kab, Mathilde Touvier, Xavier Jouven, Marcel Goldberg, Marie Zins, Jean-Philippe Empana

**Affiliations:** a Université Paris Cité, INSERM, PARCC (Paris Cardiovascular Research Center), Integrative Epidemiology of Cardiovascular Disease (Team 4), Paris, France; b Université Paris Cité, Paris Saclay University, Université de Versailles Saint-Quentin-en-Yvelines, Institut National de la Santé et de la Recherche Médicale (INSERM), UMS 011 Population-based Cohorts Unit, France; c Nutritional Epidemiology Research Team (EREN), Sorbonne Paris Nord University, Inserm U1153, Inrae U1125, Cnam, Epidemiology, and Statistics Research Center - University of Paris (CRESS), Bobigny, France

**Keywords:** Cardiovascular health, Preventive medicine, Epidemiology, Population health, Health disparities

## Abstract

**Background:**

In 2022, the previously American Heart Association (AHA) life's simple 7 score (range 0 to 14) measuring cardiovascular health (CVH) has been updated by adding sleep health and providing more granularity to the score (range 0 to 100) to measure the so-called Life's Essential 8 (LE8) score. However, the distribution of the LE8 score in nationwide representative US and non-US populations is scarce. The present study quantifies LE8 score distribution and identifies determinants of high CVH (80–100 points) in French adults.

**Methods:**

CONSTANCES is a nationwide French cohort study that randomly recruited participants aged 18 to 69 years in 24 participating health examination centers in 21 French “*départements*” in different regions of France between 2012 and 2019. Design weights for age class, sex, socio economic status, and examination center/region were applied to represent the source population. LE8 score was quantified using inclusion data on eight CVH metrics. The prevalence estimates were age-standardized directly using the 2022 EU 28 population. Mixed effects multivariable linear and logistic regression models identified key LE8 score determinants.

**Results:**

The study included 191,335 participants free of prior cardiovascular disease, with an average age of 46.48 years (SD 13.41) and 54 % women, representing 45.17 million individuals aged 18–69 in France. The overall mean LE8 score was 66.11 (68.92 in women vs. 62.79 in men, *p* = 6.875e^−7^), 13.21 %, 76.81 %, and 9.43 % achieved high (≥ 80 points), moderate (50–79 points), and poor (< 50 points) LE8 levels, respectively. Diet had the lowest mean score (41.50), while blood glycemia had the highest mean score (95.50). Mixed effects multivariable regression models identified younger age, womanhood, high educational attainment, self-employment, or managerial positions, not living with a partner, fewer depressive symptoms, lower alcohol consumption, rural residence, less socioeconomic deprivation, and absence of CVD family history as predictors of higher LE8 scores.

**Conclusions:**

Only 13.21 % of adults in France achieved a high LE8 score (≥ 80 points), and disparities related to individual and contextual socio-demographic factors and mental health were identified. The findings further underscore the importance of timely implementation of effective and personalized primordial prevention strategies.

## Graphical abstract

**Figure:**
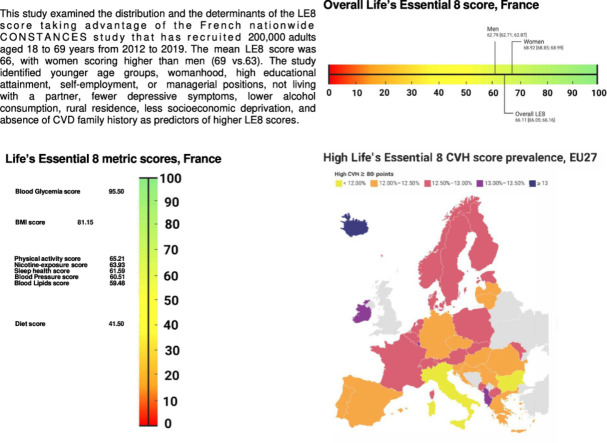

## Introduction

Despite demonstrated benefits of primary prevention strategy and public health decisions such as smoking ban to reduce the incidence of cardiovascular disease (CVD) over decades in high income countries [1] the burden of CVD remains substantial worldwide. CVD represent one-third of annual mortality in 2019 [2] is the first cause of Disability-Adjusted Life Years (DALY) in individuals over 50 old individuals and the second cause of DALYs in all ages [3] Economically, CVD healthcare management cost €282 billion in the EU 27, representing 10.6 % of total expenditure in 2021 [4] only around 3 % of which is allocated to primary care.

In this context, additional preventative strategies of CVD are urgently needed. Primordial prevention presents as a complementary prevention strategy that aims to prevent the onset of risk factors in the first place to promote and preserve a so-called ideal/optimal cardiovascular health (CVH) in the population [5] To this end, the American Heart Association (AHA) developed CVH scores, enabling population CVH monitoring [6] The Life's Essential 8 (LE8) CVH score ranges from 0 to 100 points. It includes sleep health as an eighth metric and consolidates the CVH metric criteria of the precedent Life's Simple 7 (LS7) score [7]

Higher CVH scores are associated with reduced CVD incidence, all-cause and CVD mortality outcomes [8,9] as well as non-CVD outcomes [10,11] Similarly, CVH improvement has been related to lower CVD risk or CVD mortality [12,13] and non-CVD outcomes [14,15] even for those starting with poor CVH level. Despite such evidence, the prevalence of ideal LS7 scores, i.e., 5 or 6 to 7 ideal LS7 metrics, remains below 20 % at best in adults [16] Furthermore, nationwide LE8 score prevalence estimates only derive from one single study conducted in the US population using the National Health and Nutrition Examination Survey (NHANES) [17] Also, the knowledge of LE8 score determinants are key to inform on primordial prevention implementation, but such data are currently lacking. Thus, the study's objectives were to evaluate CVH status and identify LE8 score determinants in a non-US nationwide study population involving 18 to 75-year-old individuals in France.

## 2 Methods

The CONSTANCES study is a population-representative epidemiological cohort study investigating chronic disease outcomes, healthy aging, and their occupational and social determinants [18,19] From 2012 to 2020, the cohort recruited randomly selected individuals 18 years and older from the French National Social Security database. Design weights accounting for age group, sex, socioeconomic status (SES), and region were applied to represent the source population [18,19] The participants attended 1 of the 26 participating health examination centers in 21 “*départements*” distributed over the French European territory, where they underwent physical examination and laboratory tests [18] After detailing all procedures and study objectives, all participants gave informed consent. The CONSTANCES Cohort was authorized by the National Data Protection Authority (Commission Nationale de l'Informatique et des Libertés, CNIL), n°910,486/n°1881,675), and has received favorable opinions from the National Council for Statistical Information (Conseil National de l'Information Statistique, CNIS), the National Medical Council (Conseil National de l'Ordre des Médecins, CNOM), and 0the Institutional Review Board of the French National Institute of Health and Medical Research (Institut National de la Santé et de la Recherche Médicale, INSERM).

### 2.1 LE8 score definition and measurements

The LE8 score computation was guided by the AHA-set criteria [7] The LE8 score included 8 components measuring diet, physical activity, nicotine exposure, Body Mass Index (BMI), sleep health, blood pressure, blood lipids, and blood glucose. The score for each of the eight component metrics ranges from 0 to 100. The overall LE8 was the average of all component scores, ranging from 0 to 100. The LE8 score was categorized as low (0–49), moderate (50–79), or high (80–100) [7,17] For participants on anti-hypertensive or anti-hyperlipidemic medications, 20 points were subtracted from their BP or blood lipid scores, respectively. Supplemental Methods Table M1 shows LE8 metric cut-offs.

Data collected in Constances included a food frequency questionnaire obtained weekly intakes of fruits/vegetables, soda and sweetened beverages, fish, whole grains, low-fat dairy products, and red meat. Physical activity was self-reported by a structured 6-point scale questionnaire on off-work physical activity from 0 (physically inactive/sedentary) to 6 (highly active) [20] and weekly duration of regular sports practicing (excluding trips, do-it-yourself projects, gardening, and housework) over the past 12 months. Current tobacco smoking status or period since quitting if former smoker were self-reported at inclusion. BMI was calculated as weight in kilograms divided by height in meters squared (kg/m2). Weight and height were measured in health examination centers by a trained nurse. Weight was measured using a nonautomatic weighing instrument based on the International Organization of Legal Metrology guidelines (OIML R 76–1, Edition 2006). Standing height was measured with a fixed stadiometer to the nearest 0.1 cm. Sleep health was computed from a self-reported sleep duration questionnaire in hours per night. BP was measured using an automated oscillometric sphygmomanometer (OMRON 705 CP-II/ OMRON 705IT) after a 5-minute rest in a supine position. A measurement was made for each arm, and a third was made for the reference arm with the higher systolic and diastolic BP values. The average value was used for systolic or diastolic BP. Total and high-density lipoprotein cholesterol, triglycerides, and fasting blood glucose were measured by standardized tests (COBAS Integra 400 Plus). Hemoglobin A1c was self-reported.

### 2.2 Covariates and stratification variables

Age groups were based on harmonized French National Institute of Statistics and Economic Studies (INSEE) and EUROSTAT 12 age categories ranging from 18 to 75 years. Sex was self-reported at inclusion as female or male. Residence type was classified into rural or urban according to the 2012 French census at the commune level. Socio-professional status was coded as 1) employee/manual worker, 2) self-employed/managerial, and 3) other to indicate employment status. Educational attainment was coded as 1) less than high school, 2) ≤ 2 years after high school, and 3) >2 years after high school. Cohabitation status was determined from responses to the question, “In the home where you live more often, do you live as a couple?” “yes/no”. Depressive symptomatology was assessed using the 20-item Center for Epidemiological Studies Depression score (CES-D). The Alcohol Use Disorders Identification Test (AUDIT) score assessed alcohol use modality with a cut-off ≥ 8, indicating detrimental alcohol use. The EPICES score (Evaluation of Deprivation and Inequalities in Health Examination Centres) estimates individuals’ material and psychosocial deprivation based on an 11-item score, varying from 0 to 100, with higher values indicating greater deprivation [21] CVD Family history was defined by reported known CVD in first-degree family members (e.g., parents or siblings). Geographic site was self-allocated “Where are you from? (1) Metropolitan France, (2) Overseas France, (3) Europe, (4) North Africa, (5) Sub-Saharan Africa, (6) Asia, (7) Other.”

### 2.3 Statistical analyses

The prevalence estimates were stratified by age group and sex. LE8 prevalence estimates were directly age-standardized using the EU 28 population as a reference population as of January 1st, 2022. Refer to Supplementary Methods for further methodological details and tabulated age-specific LE8 score prevalences (Supplemental Methods Table M2 and Supplemental Figure S2). Mixed-effects multivariable linear or logistic regression models were used to estimate the associations between demographic and socioeconomic health determinants and LE8 score outcomes. Random intercepts were included for geographic site and examination center. For logistic regression, odds ratio of having low or intermediate LE8 score (< 80 points) was the reference, and high LE8 score (≥ 80 points) was the outcome. The models were adjusted for age group, sex, educational attainment, employment type, cohabitation status, AUDIT-C score, CES-D score, residence type, EPICES score, and CVD family history. Missing CVH metrics (from 3745 (2.21 %) for the BMI metric to 23,435 (29.87 %) for the diet metric) and missing covariates (from 6 (0.004 %) for residence type to 46,116 (27.22 %) for employment status) were handled by multiple imputations by chained equations from 50 imputation datasets, using Rubin's rule. The preventive population fractions (PPF) were calculated using published hazard ratio estimates for CVD events associated with moderate or high LE8 levels compared to low level and applied to LE8 scores prevalence in the current study [22] assuming comparability of CVD incidence rates in the two populations.

### 2.4 Sensitivity analyses

The LE8 score level prevalences were estimated using sample design weights and complete case analysis. To assess LE8 and LS7 score levels qualitative agreements, their corresponding levels were cross-evaluated. All tests were two-sided, with a confidence interval level of 95 % and an alpha threshold of 0.05. The analyses were conducted using R version 4.1.2 and Stata version 17. (Stata Corp LLC Texas, US).

## 3 Results

**Fig. 1:**
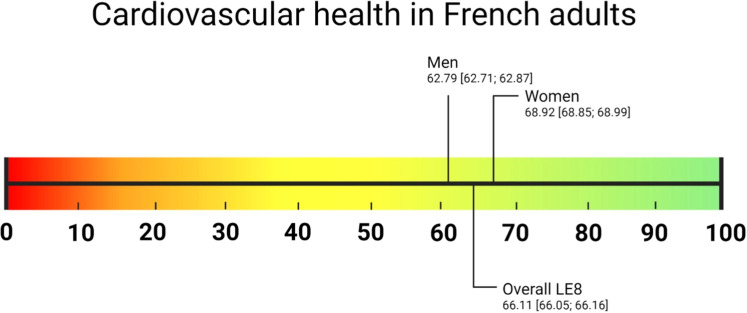
Life's Essential 8 score distribution in French adults.

**Fig. 2:**
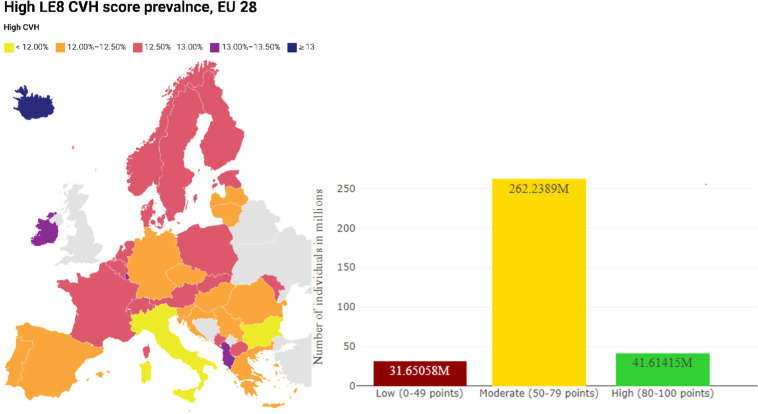
Age-standardized Life's Essential 8 score in EU 28 countries. Plot showing expected high CVH score prevalence (left panel) and distribution of low, moderate and high CVH scores in EU 28 (right panel). Life's Essential 8 score (LE8) ranges from 0 to 100 and is composed of eight cardiovascular health metrics: diet, physical activity, nicotine exposure, Body Mass Index, sleep health, blood glycemia, blood pressure, and blood lipids. LE8 CVH levels: Low LE8 (0–49 points), intermediate (50–79 points), and high (80–100 points). Age-standardized LE8 level prevalences and the expected number of individuals by CVH level were estimated using the EU 28 population as of January 1st, 2022.

**Fig. 3:**
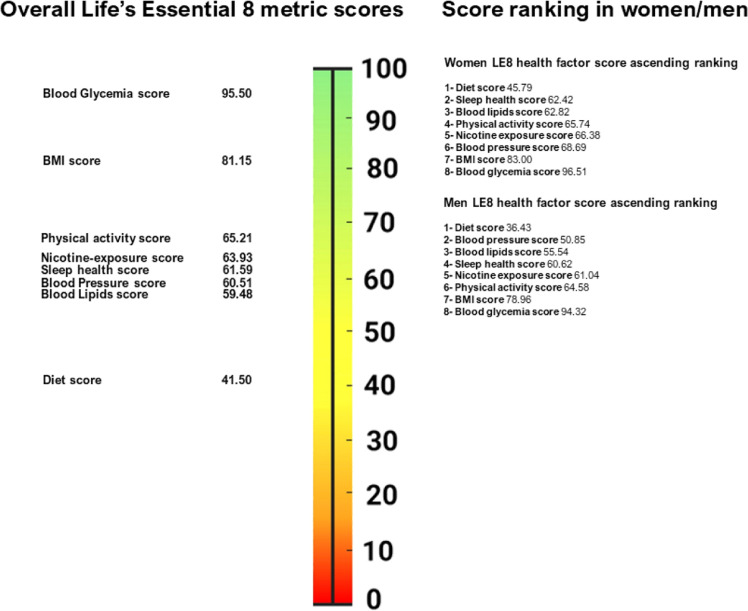
Life's Essential 8 (LE8) health factor score ranking overall, in women and in men. Each of the eight component metric scores ranged from 0 (lowest) to 100 (highest).

The study sample includes 191,335 participants without prevalent CVD, mean age 46.48 years (SD 13.41), 54 % women (Supplemental Figure 1), representing 45.17 million 18–69 old individuals in France. The overall LE8 score mean was 66.11, higher in women than in men (68.92 vs. 62.79, Fig. 1). Table 1 reports the main study sample demographic and socioeconomic characteristics according to low, moderate, and high LE8 score levels. As reported in Fig. 2, the unweighted prevalence of high (≥ 80 points), moderate (50–79 points), and low (< 50 points) LE8 score levels was 13.21 %,76.81 %, and 9.98 %, while the corresponding age-standardized estimates for the EU 28 population were 12.40 %, 78.16 %, and 9.43 %. The age-specific prevalence estimates by age group are reported in Supplemental Methods Table M2 and Supplemental Figure S2. Fig. 3 shows mean scores in women or men for each LE8 metric, which ranges from 41.50 [41.38; 41.61] for diet to 95.50 [95.42; 95.58] for glycemia. The overall mean sleep health score was 61.59, be it 62.42 in women, and 60.62 in men. Health factors such as diet, BMI, blood lipids, and blood pressure scores were lower in men than women; the ranking of the metric also differed by sex, i.e., BP score is the 2nd lowest in men, while sleep health score was the 2nd lowest in women.

**Table 1:** Sample characteristics by Life's Essential 8 (LE8) score levels in the CONSTANCES Study.

	Low LE8 (0-49)	Moderate LE8 (50-79)	High LE8 (80-100)	Mean LE8 (SD)	P for trend
Mean **Age** (SD)	50.18 [50.01; 50.34] (11.84)	46.96 [46.89; 47.02] (13.51)	40.95 [40.80; 41.11] (12.30)	-	< 0.05
**Men** (n=87,782, 45.88%)	14.39% [14.16; 14.63]	78.41% [78.13; 78.68]	7.20% [7.03; 73.75]	62.79 [62.71; 62.87] (11.98)	< 0.05
**Women** (n=103,553, 54.12%)	6.24% [6.10; 6.39]	75.46% [75.20; 75.72]	18.30% [18.06; 18.54]	68.92 [68.85; 68.99] (11.79)	
**Rural** (n=39,671, 20.73%)	12.20% [11.88; 12.52]	77.14% [76.73; 77.55]	10.66% [10.36; 10.97]	64.55 [64.43; 64.67] (12.33)	< 0.05
**Urban** (n=151,664, 79.27%)	9.40% [9.26; 9.55]	76.72% [76.51; 76.94]	13.87% [13.70; 14.05]	66.52 [66.46; 66.58] (12.22)	
**Manual worker/employee** (n=143,436, 74.97%)	9.78% [9.62; 9.93]	76.46% [76.24; 76.68]	13.76% [13.58; 13.94]	66.32 [66.26; 66.39] (12.30)	< 0.05
**Self-employed/managerial** (n=39,854, 20.83%)	10.19% [9.90; 10.50]	78.66% [78.25; 79.06]	11.15% [10.84; 11.46]	65.50 [65.38; 65.62] (11.93)	
Other (n=8,045, 4.20%)	12.55% [11.85; 13.30]	73.91% [72.94; 74.86]	13.54% [12.81; 14.30]	65.29 [65.00; 65.57] (13.07)	
**Less than High Schoo**l (n=44,347, 23.18%)	18.07% [17.71; 18.43]	77.11% [76.72; 77.50]	4.82% [4.63; 5.02]	60.87 [60.75; 60.98] (12.00)	< 0.05
**< Two Years After High School** (n=34,293, 17.92%)	11.87% [11.53; 12.21]	79.02% [78.59; 79.45]	9.11% [8.81; 9.42]	64.25 [64.13; 64.38] (11.82)	
**≥ Two Years After High School** (n=112,695, 58.90%)	6.23% [6.09; 6.37]	76.02% [75.77; 76.27]	17.75% [17.53; 17.98]	68.73 [68.67; 68.80] (11.73)	
**Cohabitation** (n= 140,350, 73.35%)	9.68% [9.52; 9.83]	77.31% [77.09; 77.53]	13.01% [12.84; 13.19]	66.14 [66.08; 66.20] (12.15)	< 0.05
**No cohabitation** (n= 50,985, 26.65%)	10.82% [10.55; 11.09]	75.44% [75.06; 75.81]	13.75% [13.45; 14.05]	66.03 [65.92; 66.13] (12.58)	
Mean **CES-D score**, range 0-60, (SD)	13.84 [13.70; 13.99] (10.28)	10.79 [10.74;10.83] (8.53)	9.11 [9.02; 9.20] (7.54)	-	< 0.05
Mean **AUDIT-C score**, range 0-40, (SD)	6.62 [6.55; 6.68] (4.48)	5.67 [5.65; 5.69] (3.37)	4.96 [4.92; 4.99] (2.62)	-	< 0.05
Mean **EPICES score**, range 0 least deprived-100 most deprived, (SD)	36.08 [35.80; 36.36] (19.82)	26.30 [26.22; 26.38] (15.37)	21.98 [21.84; 22.12] (11.42)	-	< 0.05

Life's Essential 8 score (LE8) ranges from 0 to 100 and is composed of eight cardiovascular health metrics: diet, physical activity, nicotine exposure, Body Mass Index, sleep health, blood glycemia, blood pressure, and blood lipids. Values prevalence and 95 % confidence intervals. Age Groups were classified according to the INSEE categories. Residence Type: Rural or urban designation from the 2012 census at the commune level. EPICES Score: 11-item score assessing material and psychosocial deprivation, range (0–100), with higher scores indicating greater deprivation. Households under 60 % of the national median income were considered below poverty. Depressive symptoms were assessed using the 20-item Center for Epidemiological Studies Depression Scale (CES-D) score, and depressive symptomatology was defined as ≥ 20 for women and ≥16 for men. The Alcohol Use Disorders Identification Test (AUDIT) score assessed alcohol use modality with a cutoff of ≥ 8, indicating detrimental alcohol use. Family History of CVD was declared in first-degree relatives. P-values are for Jonckheere-Terpstra test for numerical variables and Cochran-Armitage test for categorical variables.

**Fig. 4:**
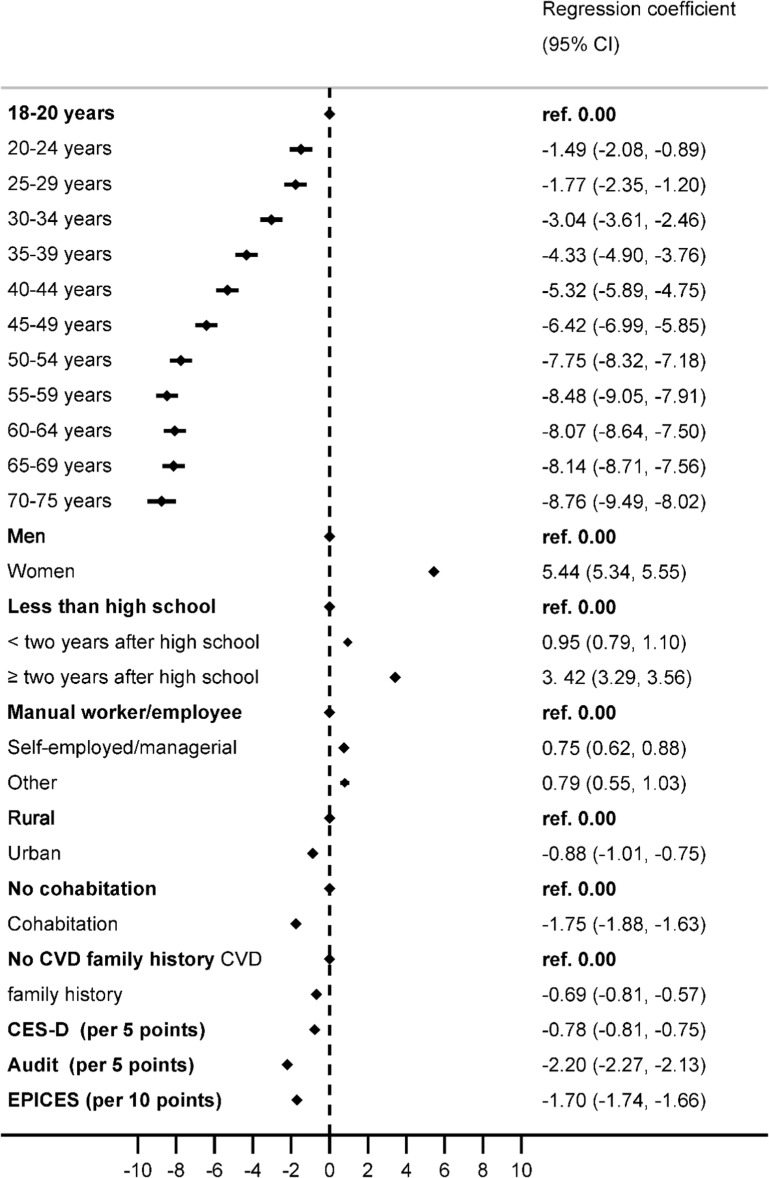
Determinants of Life's Essential 8 cardiovascular health score. Regression coefficients and their 95 % confidence intervals were estimated by mixed linear regression analysis with random intercepts on geographic site and examination center. Outcome Life's Essential 8 score (0–100 points). Values prevalence and 95 % confidence intervals. Age groups were classified according to the INSEE categories. Depressive symptoms were assessed using the 20-item Center for Epidemiological Studies Depression Scale (CES-D) score, and depressive symptomatology ranging from 0 to 60. The Alcohol Use Disorders Identification Test (AUDIT) score assessed alcohol use modality, ranging from (0–40). Residence Type: Rural or urban designation from the 2012 census at the commune level. EPICES Score: 11-item score assessing material and psychosocial deprivation, range (0–100), with higher scores indicating greater deprivation. CVD Family History was reported in first-degree relatives.

Mixed-effects multivariable linear regression model (Fig. 4) identified younger age, womanhood, high educational attainment, self- or managerial employment, non-cohabitation, less depressive symptoms, less excessive alcohol consumption, rurality, lower socioeconomic deprivation, and absence of CVD family history to be associated with a higher LE8 score. Consistent findings were obtained in logistic regression analysis for high CVH (≥ 80 points) (Supplemental Figure S3).

As shown in Supplemental Table S2, 81 % of CVD events could be avoided if all the participants would achieve a high LE8 score, 68 % for a shift from moderate to high LE8 score, and 14 % for a shift from currently 13 % to 20 % with a high LE8 score, respectively.

### 3.1 Sensitivity analyses/robustness checks

The sample-design-weighted mean LE8 score was 64.66 [64.54; 64.78]. Compared to the unweighted analysis (Supplemental Table S1), the weighted prevalence of low LE8 was higher (12.57 % vs. 9.98 %) while the weighted prevalence of high LE8 was lower (11.35 % vs. 13.21 %). Furthermore, the prevalence of high LE8 was higher in complete case analysis than in the main unweighted analysis (16.65 % vs. 13.21 %).

Cross-examination of LE8 and LS7 score levels is reported in Supplemental Figure S4. The strongest concordance was observed for the low level of CVH score (97.48 % [97.24; 97.69]). Instead, moderate and high LE8 score corresponded to 45.16 % of poor and 56 % of moderate LS7 score, respectively.

## 4 Discussion

The National Health and Nutrition Examination Survey (NHANES) is the sole prior nationwide representative study evaluating the prevalence of LE8 and this was conducted in the US population. To our best knowledge, this is the first non-US nationwide study evaluating the LE8 score prevalence in French adults. The present study also extends the results of the NHANES by providing the individual and contextual determinants of the LE8 score. While the overall mean LE8 score was consistent between the two studies (66.11 in the NHANES vs. 64.70 in the current study), the unweighted prevalence of high LE8 score was lower in the current study (13.21 % vs. 19.60 %) [17] Furthermore, compared to the NHANES, physical activity, BMI, and blood glycemia scores were higher whereas nicotine exposure, sleep health, blood pressure, and blood lipid scores were lower in the present study, the greatest difference being for the sleep health score (61.59 vs 84.70). Diet scores did not differ between the two studies. Other estimates of the LE8 score distribution derive from non-representative samples. The selected samples of the UK Biobank (27 % of the initial sample size) and the Swedish CArdioPulmonary bioImage (SCAPIS) studies, recorded high CVH prevalences of 18.9 % and 23.9 %, respectively [22] ^[^23] Mean LE8 scores were 70.0 in the China-PAR cohort and 64.6 in the Kailuan Study, with high LE8 prevalences of 23.30 % and 8.00 %, respectively [24]

Regarding LE8 score sex disparities, high LE8 score was almost three time higher in women compared to men (18.30 % vs. 7.20 %) aligning with previous findings on LS7 and LE8 score distributions [25,26] Nonetheless, adopting an intersectional analysis framework, despite its complexity, may more accurately reflect the intricate interplay of biological, socioeconomic, occupational factors [27] and preventive healthcare access and utilization as CVH determinants [28] For both men and women, diet was the lowest metric score, while blood pressure (BP) was the second lowest for men and sleep health was the second lowest for women. Furthermore, CVH disparities by sexual minority status (SM), especially in SM women [29] and rural SM men, were reported [30] suggesting that CVH determinants include sexual orientation and sexual minority status. Therefore sex and gender-inclusive CVD prevention strategies would serve to better identify and address potentially underreported or unobserved CVH disparities by sex and/or gender [31]

Individuals with higher CES-D scores indicating higher likelihood of having depression had lower LE8 score, a consistent finding with previous literature [32] This is coherent with the fact that individuals with more frequent depressive symptoms or with depression diagnosis have poorer lifestyle [32] poor adherence to pharmacological treatments [33] and are less likely to healthcare access [34]

Urban regions were associated with lower CVH scores than rural ones. Systematic evidence on rural/urban CVH disparities is scarce [35] Increasing population density and reduced walkability index in urban living environments have been deemed obesogenic and diabetogenic, which may be explained by reduced physical activity and increased consumption of processed and unhealthy food [[36], [37], [38]] National and city-level urban health policies aim to address environmental CVH determinants [39] and include efforts to reduce air, light, and noise pollution [40] ensure water and food security, plan for sustainability, enhance extreme weather resilience, and improve access to green and blue spaces [39] Furthermore, a smart connected city infrastructure may enable personalized CVH real-time personalized CVH monitoring through wearable sensors, and Internet of Things (IoT) to provide sustainable citizen-engaged data-driven CVD prevention.

Diet recorded the lowest average scores, underscoring the need for interventions to promote healthy eating and combat food insecurity [41] Establishing dietary guidelines and policies to ensure access to affordable fresh and healthy food are some of the obstacles to achieving higher CVH [42] Consequently, more research is needed to quantify the impact of non-nutritional dietary factors [43] front-of-package labeling [44] and commercial determinants of LE8 diet metric [45,46]

Data on primordial prevention implementation are currently lacking in France, whereas our simulation analysis underlines its potential for strong public health impact on CVD. Indeed, when emulating a realistic scenario envisaging a shift from currently 13 % to 20 % of the population with a high CVH score ≈14 % of CVD events could be prevented. Furthermore, CVH scores declined progressively from 18–20 to 55 years, plateauing thereafter, as previously reported [17,47] Such findings support lifelong primordial prevention implementation with interventions adapted to age, location, accessibility and life transition periods [48] For instance, schooling has been suggested as an advantageous early primordial prevention implementation period [49,50]

Despite a sufficiently powered sample size, a nationwide design, and availability of the eight CVH metrics data, the CONSTANCES study has several limitations. The LE8 diet metric was incomplete since information on sodium or dietary fiber was unavailable. HbA1c level relied on self-reported data since HbA1C was only measured in 1 % of the participants. Still, self-reported HbA1c has been validated in previous epidemiological studies [51,52] Despite the use of standardized operating procedures, health behaviors were self-reported so that classification biases cannot be excluded. It is also safer to assume that if selection effect exist, it may have favored participants with healthier behaviors, thus the weighted LE8 prevalence estimates may be closer to that of the target population. Data for individuals in overseas territories were unavailable, preventing the evaluation of CVH in those departments. The current study was conducted in a high-income country with universal health care insurance, precluding generalizing the findings to resource-limited public health settings.

## 5 Conclusions

Despite having a free access to health care nationwide, only 13 % of the adult population is currently achieving an optimal cardiovascular health as defined by the LE8 score. The study therefore identified missed opportunities for better cardiovascular health and advocates for shifting from a mainly care model to a more balanced care and preventive model in France. The findings highlight the need for effective and timely implementation of primordial prevention strategies. Such interventions should account for individual and contextual factors and, require transdisciplinary expertise in addition to the population concerned.

## CRediT authorship contribution statement

**Omar Deraz:** Writing – review & editing, Writing – original draft, Visualization, Validation, Software, Methodology, Funding acquisition, Formal analysis. **Sofiane Kab:** Data curation. **Mathilde Touvier:** Resources. **Xavier Jouven:** Resources. **Marcel Goldberg:** Writing – original draft, Methodology, Data curation, Conceptualization. **Marie Zins:** Resources. **Jean-Philippe Empana:** Writing – original draft, Supervision, Funding acquisition, Conceptualization.

## Declaration of competing interest

The funders had no role in the study conceptualization, data analyses, manuscript drafting, or the decision to publish the study and publisher choice. No further disclosures are made.

## Appendix Supplementary materials

Image, application 1
