# Supplementary material for: Life's Essential 8 cardiovascular health status of 18–69-year-old individuals in France
Source: Am J Prev Cardiol. 2025 Mar 30;22:100981. doi: 10.1016/j.ajpc.2025.100981 (PMC12003004; doi:10.1016/j.ajpc.2025.100981)
Supplement: Supplementary file 1 [file mmc1.docx]

**Supplemental material**

**Supplemental Tables**

Supplemental Table S1. Complete case and weighted Life’s Essential 8 score prevalence estimates.

|  | **Low LE8 (%)** | **Moderate LE8 (%)** | **High LE8 (%)** | **Mean LE8 Score (SD)** |
| --- | --- | --- | --- | --- |
| **Unweighted estimates in main analysis**  **(n=191,335))** | 9.98%  [9.85; 10.12] | 76.81%  [76.62; 77.00] | 13.21%  [13.06; 13.36] | 66.11 [66.05; 66.16]  (12.27) |
| **Age-standardized estimate** | 12.40% | 78.16% | 9.43% | - |
| **Complete case estimate**  **(n= 98,634)** | 9.31%  [9.13; 9.49] | 74.05%  [73.77; 74.32] | 16.65%  [16.42; 16.88] | 67.15 [67.07; 67.23]  (12.70) |
| **Sample weighted estimates**  **(n= 102,305)** | 12.57%  [12.23; 12.90] | 76.08%  [75.67; 76.48] | 11.35%  [11.10; 11.63] | 64.66 [64.54; 64.78]  (12.30) |

LE8: Life’s Essential 8 score (range 0-100). LE8 categories were low (0-49 points), moderate (50-79 points), and high (80-100 points). Age-standardization of LE8 score level prevalences was conducted for the EU 28 population as of January 1^st^, 2022.

Supplemental Table S2. Averted CVD cases for CVH promotion scenarios in France and EU 28 countries.

| **CVH promotion scenario** | **Population preventive fraction (PPF)** | **Annual preventable CVD cases (France)** | **Annual preventable CVD cases (EU 28)** |
| --- | --- | --- | --- |
| 1- All participants achieve a high LE8 level | 80.79% | 945 165 | 7.02 million |
| 2- From moderate to high LE8 level | 67.94% | 794 832 | 5.90 million |
| 3- From currently 13% to 20% with high LE8 level | 13.79% | 343 610 | 1.20 million |

The estimates were re-produced using EU 28 population on January 1^st^, 2022.

**Supplemental figures**


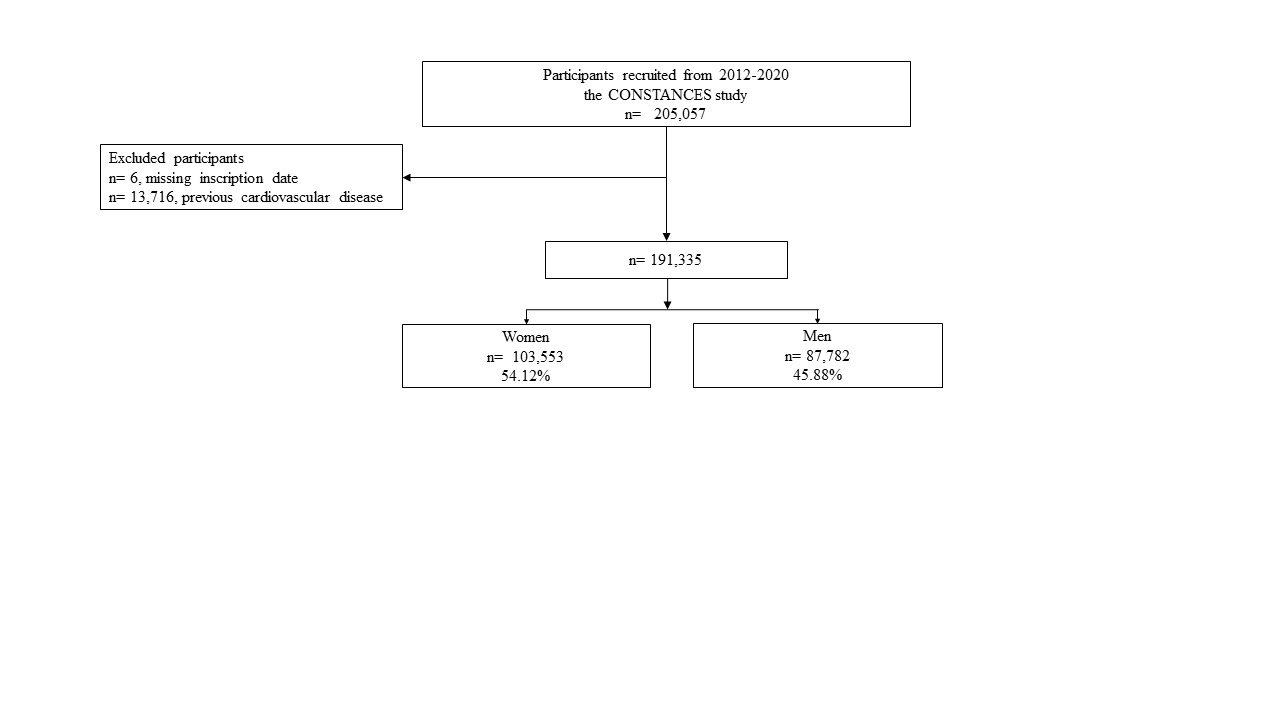
Supplemental figure S1. Study sample flowchart.


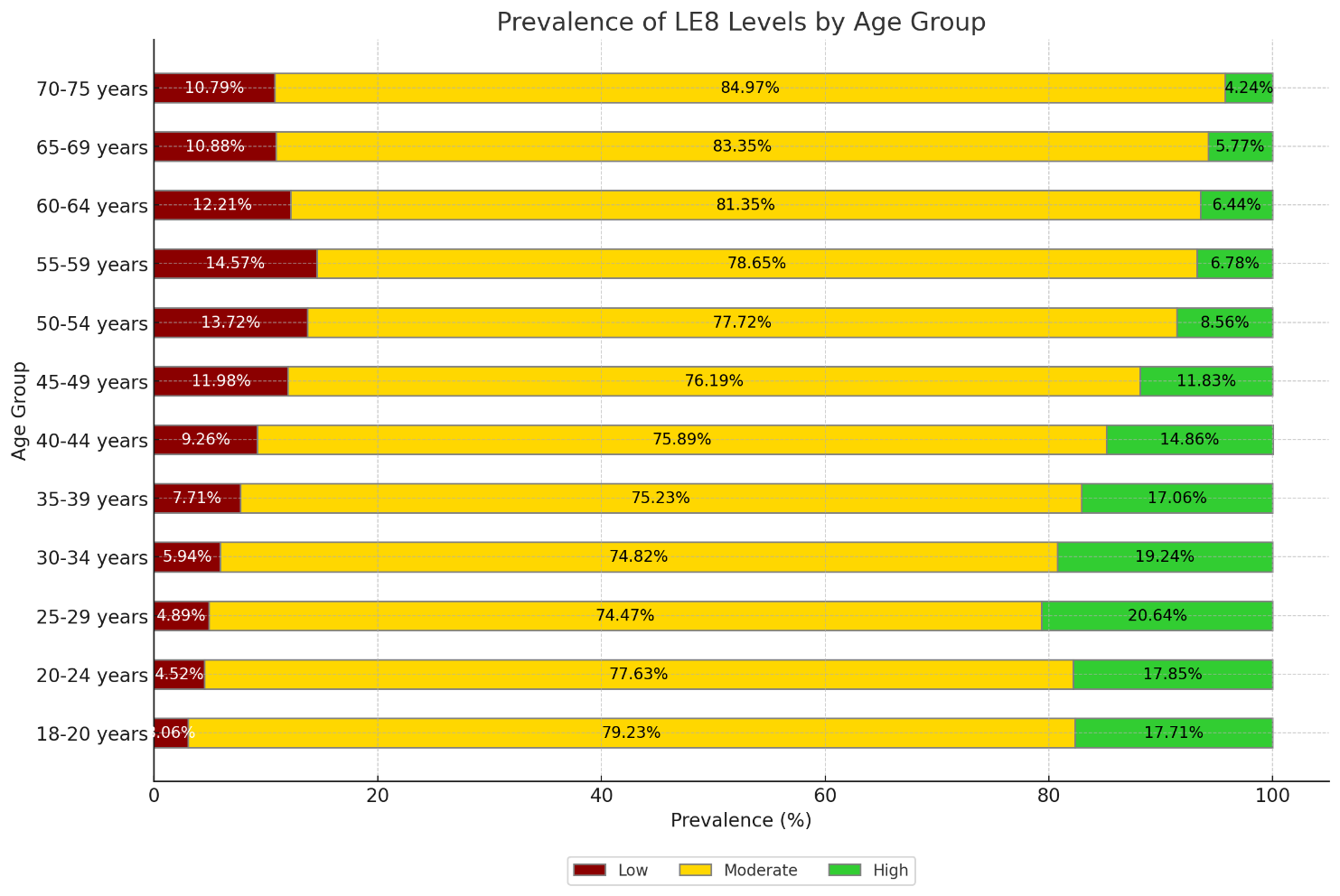
Supplemental Figure S2. Expected age-specific LE8 score level prevalences for the EU 28 population.

LE8: Life’s Essential 8 score. Low LE8 (0-49 points), intermediate (50-79 points), and high (80-100 points). Expected age-specific LE8 score level prevalences for the EU 28 population as of January 1^st^, 2022.



Supplemental Figure S3. Adjusted multivariable mixed-effects logistic regression model estimates for determinants of high LE8 score (80-100 points).

Outcome high Life’s Essential 8 score (80-100 points) compared to low (0-49 points) or intermediate levels (50-79 points). Values prevalence and 95% confidence intervals. Age Groups were classified according to the INSEE categories. Depressive symptoms were assessed using the 20-item Center for Epidemiological Studies Depression Scale (CES-D) score, and depressive symptomatology was defined as ≥20 for women and ≥16 for men. The Alcohol Use Disorders Identification Test (AUDIT) score assessed alcohol use modality with a cutoff of ≥8, indicating detrimental alcohol use. Residence Type: Rural or urban designation from the 2012 census at the commune level. EPICES Score: 11-item score assessing material and psychosocial deprivation, range (0-100), with higher scores indicating greater deprivation. CVD family history was self-reported for first-degree relatives.

**
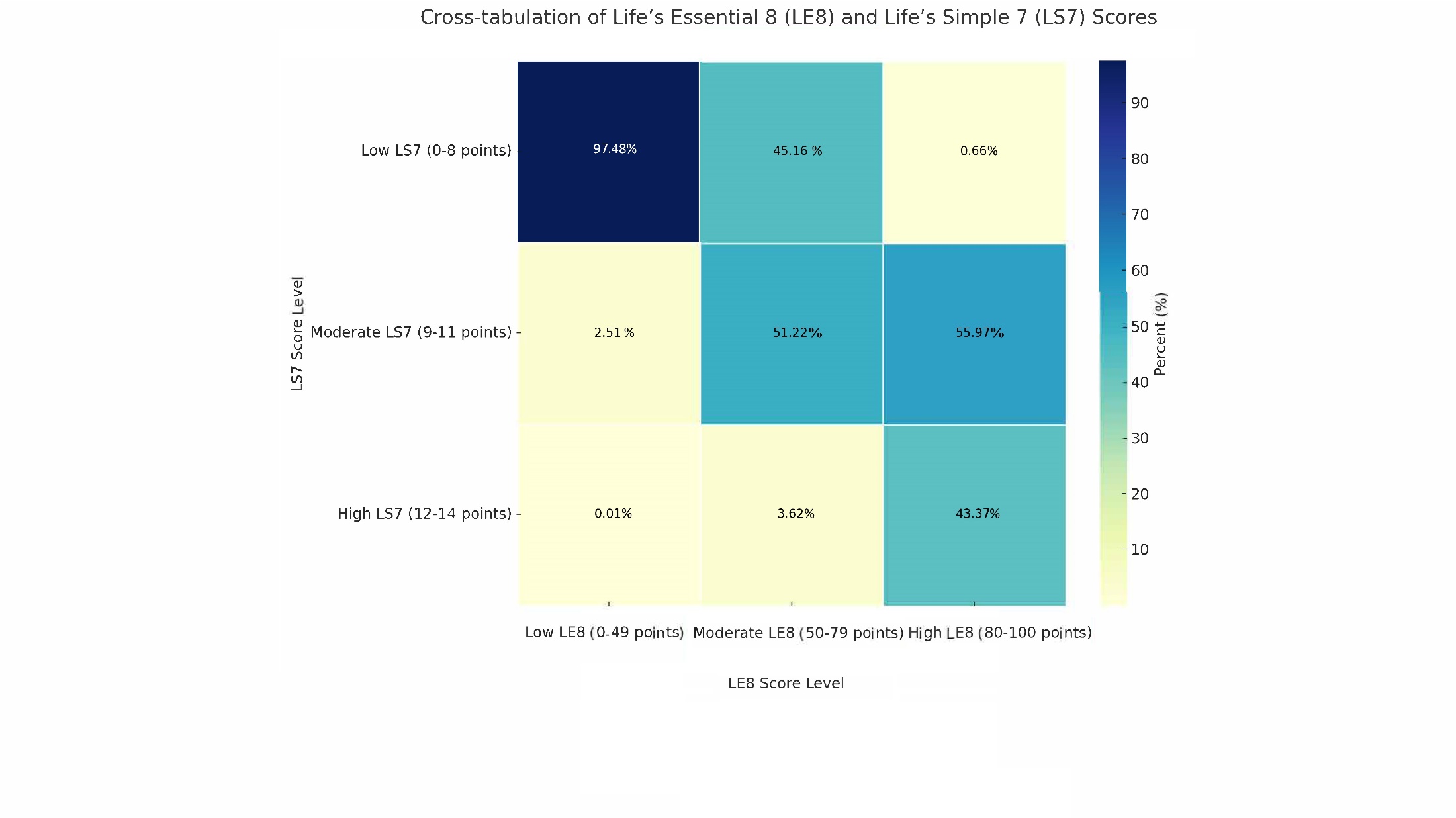
**Supplemental Figure S4. Cross-tabulation of Life’s Essential 8 (LE8) and Life’s Simple 7 (LS7) scores.

LS7: Life’s Simple 7 cardiovascular health score (range 0-14). LE8: Life’s Essential 8 cardiovascular health score (range 0-100). 0Rows represent LS7 levels (Low, Moderate, High). Columns LE8 levels (Low, Moderate, High), including the percentage of LS7 levels within each LE8 category and confidence intervals for these percentages.

**Supplemental methods**

To test for linear regression assumptions, residual plots evaluated the linearity assumption for CVH scores. Residuals versus predicted values plots evaluated heteroscedasticity. The normality assumption was tested using the Kolmogorov-Smirnov test. Logit linearity for numerical variables was tested using the Boxtid test.

For linear or logistic regression models, the Haussman test determined statistical need for random intercepts compared to fixed ones in regression models. The Variance inflation factor (VIF) assessed multicollinearity; The Bayesian Information Criterion (BIC) determined model selection, goodness of fit and statistical parsimony.

**Supplemental Methods Tables**

Supplemental Methods Table M1. Life’s Essential 8 score metrics’ definitions.

| Metric | Definition Modification |
| --- | --- |
| Diet score | Weekly intakes of fruits/vegetables, soda and sweetened beverages, fish, whole grains, low-fat dairy products, and red meat were obtained from a food frequency questionnaire.^19^ Data were unavailable for dietary fibers and daily salt consumptions at the time of analyses.  The diet score was calculated from percentiles of Dietary Approaches to Stopping Hypertension (DASH) intake percentile, ranging from 0-100.^13^  100 if ≥95^th^ percentile  80 if 75^th^-94^th^ percentiles  50 if 50^th^-74^th^ percentiles  25 if 25^th^-49^th^ percentiles  0 if 1^st^-24^th^ percentiles |
| Physical activity score | Self-reported physical activity and weekly duration of regular sports practicing (excluding trips, DIY, gardening, and housework) over the past 12 months. Physical activity scores was computed based on the equivalent of weekly minutes of moderate or high intensity off-work or sports activity, ranging from 0-100.^13^  100 if ≥150 mins  90 if 120-149 mins  80 if 90-119 mins  60 if 60- 89 mins  40 if 30-59 mins  20 if 1-29 mins  0 if 0 mins or sedentary/inactive |
| Nicotine exposure score | Tobacco smoking, inhaled electronic nicotine delivery systems (NDS) use status, and period since quitting if former smoker were self-reported at inclusion. Negative smoking status was not available. Nicotine exposure score ranged from 0-100.^13^  100 for never smokers  75 for quitters ≥ five yrs ago  50 for quitters 1-5 yrs ago  25 for either quitters < one yr ago or inhaled electronic nicotine delivery systems (NDS) users  0 for current smokers |
| Sleep health score | Self-reported sleep duration in hours per night defined the sleep health score ranged from 0-100,.^13^  100 if 7-<9 hours  90 if 9-<10 hours  70 if 6-<7 hours  40 if either 5-<6 hours or ≥10 hours  20 if 4-<5 hours  0 if <4 hours |
| Body Mass Index (BMI) score | Weight and height were measured in health examination centers by a trained nurse. BMI was calculated as weight in kilograms divided by height in meters squared (kg/m2), BMI score ranged from 0-100.^13^  100 if <25.00 kg/m^2^  70 if 25.00–<30.00 kg/m^2^  30 if 30.00–<35.00 kg/m^2^  15 if 35.00-<40.00 kg/m^2^  0 if ≥40.00 kg/m^2^ |
| Blood glucose score | Fasting blood glucose were measured by standardized tests (COBAS Integra 400 Plus®). Glycated hemoglobin (HbA1c) was self-reported. Blood glucose score ranged from 0-100.^13^  100 if no known type 2 diabetes (T2D) history, no anti-hyperglycemic medication, and fasting blood glucose (FBG) <100 mg/dL or HbA1c <5.70%  60 if no known T2D history, no anti-hyperglycemic medication and FBG 100-125 mg/dL or HbA1c 5.70%-6.40%  40 if known T2D history or anti-hyperglycemic medication and HbA1C <7.00%  30 if known T2D history or anti-hyperglycemic medication and HbA1C 7.00%-7.90%  20 if known T2D history or anti-hyperglycemic medication and HbA1C 8.00%-8.90%  10 if known T2D history or anti-hyperglycemic medication and HbA1C 9.00%-9.90%  0 if known T2D history or anti-hyperglycemic medication and HbA1C >10.00% |
| Blood pressure score | Blood pressure (BP) was measured using an automated oscillometric sphygmomanometer (OMRON® 705 CP- II/OMRON® 705IT) after a 5-min rest in a supine position. A measurement was made for each arm, and a third was made for the reference arm with the higher systolic and diastolic BP values. The average value was used for systolic or diastolic BP. BP score was quantified using systolic and diastolic BP levels in mm Hg. BP score ranged from 0-100.^13^  100 if <120/80 mm Hg  75 if 120-129 mm Hg and <80 mm Hg  50 if 130-139 and 80-89 mm Hg  25 if 140-159 mm Hg and 90-99 mm Hg  0 if ≥160 mm Hg or ≥100 mm Hg  In case of medication intake, 20 points were subtracted |
| Blood lipids score | Total and HDL cholesterol were measured by standardized tests (COBAS Integra 400 Plus®). Blood lipids score was calculated using non–HDL cholesterol (mg/dL). Blood lipids score ranged from 0-100.^13^  100 if non–HDL cholesterol <130 mg/dL  60 if non–HDL cholesterol 130-159 mg/dL  40 if non–HDL cholesterol 160-189 mg/dL  20 if non–HDL cholesterol 190-219 mg/dL  0 if non–HDL cholesterol ≥220 mg/dL  In case of medication intake, 20 points were subtracted |

Supplemental Methods Table M2. Direct standardization of LE8 score level prevalence to the EU 28 2022 population.

| **Age-group** | **LE8 score level** | **Observed sample (n)** | **Age-specific prevalence** | **EU28 population per age-group** | **Expected EU28 population** |
| --- | --- | --- | --- | --- | --- |
| 18-20 years | Low | 46 | 3.06 | 23220320 | 710542 |
|  | Moderate | 1177 | 79.23 |  | 18397460 |
|  | High | 279 | 17.71 |  | 4112319 |
| 20-24 years | Low | 332 | 4.52 | 23900313 | 1080294 |
|  | Moderate | 5624 | 77.63 |  | 18553813 |
|  | High | 1393 | 17.85 |  | 4266206 |
| 25-30 years | Low | 768 | 4.89 | 25335811 | 1238921 |
|  | Moderate | 11498 | 74.47 |  | 18867578 |
|  | High | 3432 | 20.64 |  | 5229311 |
| 30-34 years | Low | 1147 | 5.94 | 28134334 | 1671179 |
|  | Moderate | 14256 | 74.82 |  | 21050109 |
|  | High | 3922 | 19.24 |  | 5413046 |
| 35-39 years | Low | 1635 | 7.71 | 29110270 | 2244402 |
|  | Moderate | 15722 | 75.23 |  | 21899656 |
|  | High | 3858 | 17.06 |  | 4966212 |
| 40- 44 years | Low | 2244 | 9.26 | 30646596 | 2837875 |
|  | Moderate | 18173 | 75.89 |  | 23257702 |
|  | High | 3826 | 14.86 |  | 4554084 |
| 45-49 years | Low | 2559 | 11.98 | 31391394 | 3760689 |
|  | Moderate | 16135 | 76.19 |  | 23917103 |
|  | High | 2666 | 11.83 |  | 3713602 |
| 50-54 years | Low | 2715 | 13.72 | 32409024 | 4446518 |
|  | Moderate | 15268 | 77.72 |  | 25188293 |
|  | High | 1805 | 8.56 |  | 2774212 |
| 55-59 years | Low | 3003 | 14.57 | 31794915 | 4632519 |
|  | Moderate | 16117 | 78.65 |  | 25006701 |
|  | High | 1485 | 6.78 |  | 2155695 |
| 60-64 years | Low | 2483 | 12.21 | 29530602 | 3605687 |
|  | Moderate | 16442 | 81.35 |  | 24023145 |
|  | High | 1409 | 6.44 |  | 1901771 |
| 65-69 years | Low | 1964 | 10.88 | 26569096 | 2890718 |
|  | Moderate | 14976 | 83.35 |  | 22145342 |
|  | High | 1113 | 5.77 |  | 1533037 |
| 70-75 years | Low | 201 | 10.79 | 23458925 | 2531218 |
|  | Moderate | 1579 | 84.97 |  | 19933049 |
|  | High | 83 | 4.24 |  | 994658 |

LE8: Life’s Essential 8 score. Low LE8 (0-49 points), intermediate (50-79 points), and high (80-100 points). Population used for direct standardization: EU 28 age population structure on January 1^st^, 2022.
